# Editorial: Dietary management in children with immune-related diseases

**DOI:** 10.3389/fnut.2023.1185724

**Published:** 2023-03-27

**Authors:** Emilia Vassilopoulou, Alessandra Mazzocchi, Valentina De Cosmi

**Affiliations:** ^1^Department of Nutrition and Dietetics, International Hellenic University, Thessaloniki, Greece; ^2^Department of Clinical Sciences and Community Health, University of Milano, Milan, Italy

**Keywords:** immunonutrition, immunomodulation, infant, child, nutrient

This special issue of Frontiers in Nutrition entitled *Dietary Management in Children with Immune Related Diseases* hosts scientific articles that enrich scientific knowledge about the role of nutrition in modulating and enhancing immune function to prevent chronic inflammatory diseases. The role of diet in the achievement and maintenance of a healthy status is well recognized, and in recent years, evidence has been presented of a link between a well-functioning immune system and good health overall. The immune system has a central role, not only in fighting infection, but also in regulating many acute and chronic disorders.

This collection of four articles highlights the fundamental role of balanced nutrition in certain immune related conditions in childhood. In the first, Mbugi et al. conducted a cross-sectional study in three African districts with the highest prevalence of malaria and potential micronutrient deficiencies, exploring the effect of micronutrients, and in particular zinc, on the profiles of IgG subclasses in Tanzanian children aged ≤5 years. The potential for production of antibodies is influenced by micronutrient status, and the profile of both cytophilic and non-cytophilic IgG subclasses was reported to be variably influenced by the zinc status, with IgG3 being critically affected across all the groups in the age range studied. The administration of precise amounts of zinc boosts the production of protective IgG subclasses, and should be considered in malaria interventional programs for children in endemic areas.

The case study by Feketea et al. of four clinical cases proposes the term FPIPLE for a food-protein induced protein-losing enteropathy. FPIPLE is a mixed IgE and non-IgE food allergy in young infants, along with eosinophilic gastrointestinal diseases (EGID). The cases presented showed the same clinical pattern: poor weight gain, edema due to hypoproteinemia/hypoalbuminemia from enteral loss of proteins, anemia, eosinophilia, raised levels of fecal α1-antitrypsin and specific-IgE, and allergy skin prick test positive for offending foods, establishing a distinct clinical entity. Protein-losing enteropathy associated with gastrointestinal allergy has been described in adults, but data on clear diagnostic criteria in infancy have been limited (1). Diagnostic criteria are presented, with an algorithm of practical methodology for the diagnosis and management of children suspected to have FPIPLE.

The paper by Vassilopoulou et al. explores the association of food protein-induced allergic proctocolitis (FPIAP) with the maternal diet during pregnancy and breastfeeding in Greek infants. FPIAP is a transient benign condition causing colitis in infants, often presenting in the first month of life. It is a non-IgE mediated immune response to one or more foods, with inflammation in the distal colon, eosinophilic infiltration of the rectal mucosa, and blood in the feces (2). A multicenter retrospective case-control study was conducted in Greece, on mothers of infants with and without a history of FPIAP. Information was collected on the maternal diet using a validated food frequency questionnaire and the Mediterranean Diet Score Questionnaire on adherence to the Mediterranean Diet. The results showed that elements of the Mediterranean Diet may be protective against FPIAP. Frequent consumption of food home-cooked according to the traditional Mediterranean customs, wholewheat grains, fruits, fish and shellfish, and supplementation of folic acid, vitamins D, A, B-complex, and multivitamins appeared to protect against FPIAP in infancy. Conversely, a diet with a high content of fat, sugary products, salt, vegetables and dietary fiber, vitamin C and n-3 PUFA supplementation, and use of non-stick kitchenware/grills appeared to increase the risk of FPIAP. A family history of allergy was a strong predisposing factor for FPIAP.

In the fourth paper, Muratore et al. reviewed the role of nutrition in the modulation of intestinal flora, which appears to be important in the pathophysiology of the main complications of hematopoietic stem cell transplantation (HSCT) in childhood. Malnutrition of a complex and multifactorial nature is documented to affect 10–50% of children undergoing HSCT, and although it significantly affects outcomes, it continues to be largely unrecognized. The review concludes that nutritional support could be a risk- and cost-effective way of improving allo-HSCT outcomes in children.

Despite the large current body of literature related to dietary management in children with immune-related diseases, the papers in this special issue show that many areas remain unexplored regarding the correlation of dietary factors with immune status and health. Firstly, the effects of single nutrients on immune function are difficult to study, but well-designed intervention studies to investigate the effects of overall dietary patterns on the immune system represent real life conditions, and would be more feasible, and their results more reliable. Secondly, the prevalence of FPIPLE in primary care and pediatric allergy and gastroenterology practice is unknown, and better characterization of this mixed IgE and non-IgE mediated food allergy is needed. Thirdly, intervention studies during pregnancy and lactation might provide more robust evidence on the appropriate dietary interventions for mothers to prevent FPIAP in their infants. Finally, in-depth nutritional assessment is required, with evaluation of the appropriate dietary intervention to improve allo-HSCT outcomes in children.

## Author contributions

All authors listed have made a substantial, direct, and intellectual contribution to the work and approved it for publication.
